# Effect of Short-Term High-Temperature Stimuli on the Functional Response of *Trichopria drosophilae* (Matsumura)

**DOI:** 10.3390/insects14090748

**Published:** 2023-09-06

**Authors:** Qiang Chen, Jinlong Zhang, Ye Tian, Guohua Chen, Xiaoming Zhang

**Affiliations:** State Key Laboratory of Yunnan Biological Resources Protection and Utilization, College of Plant Protection, Yunnan Agricultural University, Kunming 650201, China; chenqiangcqkm@126.com (Q.C.); sasazjl@126.com (J.Z.); tianyelee@126.com (Y.T.)

**Keywords:** *Trichopria drosophilae*, *Drosophila suzukii*, biological control, temperature, functional response

## Abstract

**Simple Summary:**

Soft fruit is an economically important global fruit crop. *Drosophila suzukii* is a significant pest of soft fruit, including grape bayberry. In China, *Trichopria drosophilae* is the dominant pupal parasitoid of *D. suzukii*. In order to control the population of *D. suzukii* more effectively and reduce the use of pesticides, researchers have investigated the potential role of *T. drosophilae* in the biological control of *D. suzukii* in China. In this paper, we investigated the functional response and control potential of *T. drosophilae* on *D. suzukii* pupae at different temperatures. Our aim was to expand the range of control applications for *T. drosophilae*. We investigated the functional response and interference effect of *T. drosophilae* and found that *T. drosophilae* showed good levels of parasitic ability on *D. suzukii* pupae in the laboratory environment. At temperatures of 23 °C to 35 °C, *T. drosophilae* exhibited a type II functional response on *D. suzukii* pupae.

**Abstract:**

Researchers have previously investigated the role of *Trichopria drosophilae* as a pupal parasitoid in the biological control of *Drosophila suzukii* in China. Here, we investigated the ability of *T. drosophilae* to parasitize *D. suzukii* pupae at different temperatures. To do this, we evaluated the functional response of *T. drosophilae* to *D. suzukii* pupae at different temperatures and investigated the specific effects of density on parasitism. The results show that the parasitic functional response of *T. drosophilae* under different high-temperature stimuli is Holling type II. After processing at 29 °C, the instantaneous search rate was 1.1611; the theoretical maximum parasitic value was 20.88 at 31 °C. The parasitic efficiency decreased with increasing stimulation temperature, as the host pupa density increased from 5 to 25, and the strongest search effect occurred at 0.87 at 27 °C. The searching effect of *T. drosophilae* at each temperature fell gradually with an increase in prey density from 5 to 25. At 31 °C, the theoretical parasitic maximum of *T. drosophilae* reached a maximum of 20.88 pupae. At this temperature, when a pair of *T. drosophilae* was placed in a pupa density of 50, its actual total number of parasites was 18.60.

## 1. Introduction

The spotted wing Drosophila, *Drosophila suzukii* [Matsumura] (Diptera: Drosophilidae) was first discovered in 1916 in a strawberry orchard in the Yamanashi Prefectureof Japan [1]. *D. suzukii* was first detected in California, USA, in 2008. In Europe, *D. suzukii* was detected in Italy and Spain in 2009 and has spread rapidly; as of 2018, *D. suzukii* has spread globally to Mexico and Slovenia [2]. The host plants of *D. suzukii* are extensive; thus, the effect of this pest can cause damage to many economically important fruits, including *Myrica rubra*, *Cerasus pseudocerasus*, *Vitis vinifera*, and *Rubus corchorifolius* in China. Studies have shown that *D. suzukii* not only feeds on fruits with damaged skin or fruit that has dropped to the ground, but it also causes damage to mature or almost mature fresh fruit with soft skins. A female can directly insert her ovipositor into relatively mature or mature fresh fruit with soft skin and lay eggs directly into the pulp. The larvae subsequently feed on the pulp and then move their feeding point to the surrounding tissue, thus causing rapid decay and softening. This accelerates decay, renders the fruit inedible, and causes huge economic losses in the fruit industry [3]. According to statistics, in 2009, the damage rate of *D. suzukii* to cherry crops in California, USA, reached 20%, and the damage caused by *D. suzukii* to blueberries, raspberries, and strawberries reached 40%, 70%, and 80%, respectively [4,5]. Worryingly, the potential economic losses caused by *D. suzukii* continue to rise globally [6].

At present, *D. suzukii* is controlled mainly by chemical agents. However, the oviposition habit of *D. suzukii* and its resistance to chemical control measures resulting from the extensive and widespread use of chemical agents have led to a reduction in the efficacy of chemical control [7]. Based on the unique style of oviposition habit and the damaging characteristics of *D. suzukii*, the use of parasitic natural enemies to control *D. suzukii* is particularly promising [8]. In recent years, many countries have begun to strengthen their research on the biological control of *D. suzukii* in an attempt to make use of effective natural enemy resources and other control means to achieve sustainable development and control the occurrence of pests. Giving full play to the natural control effect of parasitic natural enemies on *D. suzukii* is the most economical and effective method to control *D. suzukii*. As one of the earliest countries in Asia to discover *D. suzukii*, China has identified more parasitic natural enemy insect resources because of its highly diverse natural geographic conditions. Of these resources, *Trichopria drosophilae* is one of the most effective parasitic wasps [9].

*T. drosophilae* belongs to the Hymenoptera: Diapriidae [10] and is an active parasite of *D suzukii* pupae. *T. drosophilae* has been shown to lay eggs directly into the host after anesthesia [11]. Many studies have proved that this ubiquitous parasitoid can successfully parasitize the pupae of *D. suzukii* in Asia, Europe, and North America [12]; furthermore, the parasitization rates of *D. suzukii* by *T. drosophilae* are high under laboratory conditions, and rearing in large numbers is feasible. In the south of Italy, Rossi Stacconi and other scholars used the local *T. drosophilae* population to control *D. suzukii* in cherry orchards; this practice significantly reduced the degree of insect damage in the orchards, which led to a 34% reduction in the damage caused by fruit insects. Collectively, these results demonstrated that the local *T. drosophilae* population could be used as a biological control method to control the economic losses caused by *D. suzukii* through field release combined with other pest control methods [13].

Studying the reproductive attributes that make parasitoids effective pest control agents, as well as the potential insect host–parasitoid interactions and behavioral ecology of parasitoids, is important for the successful application of parasitoids in biological control [14]. The reproductive success of an individual parasitoid depends on a combination of host and parasitoid interactions; in principle, it can be integrated into a host–parasitoid model to describe the temporal and spatial dynamics of host–parasitoid interactions. This type of host–parasitoid model is driven by the specific type of functional response, defined as the number of attacked hosts or prey consumed within a limited time interval in relation to host/prey density [15]. A simulation model related to host–parasitoid population dynamics is currently being developed to analyze the efficacy of *T. drosophilae* for the biological control of *D. suzukii* and to recommend further strategies for biological control.

An important component of this model is the functional response that describes the relationship between host density and the number of attacks carried out per time unit by an individual female parasitoid [16]. According to the shape of the response curve, the functional response is divided into type I (a linear increase), type II (a monotonic decelerating increase), type III (a sigmoidal increase), or type IV (dome shaped) [17]. Kacar et al. found that the type of parasitic functional response of *T. drosophilae* to *D. suzukii* and *D. melanogaster* pupae is a linear function [16]. Furthermore, parasitoid life history characteristics are known to change in response to temperature, and humidity can also alter these characteristics, but to a lesser extent, except under extreme conditions [18]. Similarly, the search and attack rates of parasitoids are known to be affected mainly by temperature [19]. Wu [20] treated *Trichogramma nigra* at 30, 33, 36, and 39 °C and four handling times of 2, 4, 6, and 8 h respectively. Analysis showed that a temperature of 30 °C increased the parasitic effect of *Trichogramma pintoi*. However, with an increase in temperature stimuli from 30 °C to 39 °C, the handling time was prolonged and the parasitic effect of *T. pintoi* was significantly inhibited; this may be related to the heat tolerance of this species. In addition, high temperature may change the functional response type of a parasitoid to the host. For example, Xie et al. [21] found that the parasitic functional response of two strains of *Trichogramma dendrolimi* to rice moth eggs changed from type III to type II with increasing temperature.

In the present study, we evaluated the functional response of *T. drosophilae* females to different densities (5, 10, 15, 20, and 25 host pupae/box) of *D. suzukii* after different temperature stimuli (23, 27, 29, 31, 33, and 35 °C). The purpose of this study was to clarify the type of parasitic functional response of *T. drosophilae* under the influence of temperature and to screen the optimal temperature and the ratio of parasitic wasps to host in the process of *T. drosophilae* expanding reproduction.

## 2. Materials and Methods

### 2.1. Insect Cultures

Colonies of *Drosophila suzukii* were derived from specimens emerging from *Myrica rubra* collected in Dianwei village, Kunming (102°9′ E, 24°8′ N), Yunnan Province, Southwest China, during the summer of 2016. The *Trichopria drosophilae* colony was derived from specimens emerging from infested fruit collected from the same location in Kunming in 2016. The stock culture of *D. suzukii* was held in incubators with a temperature of 25 ± 1 °C, a photoperiod of 16 h:8 h light/dark (L:D), and a relative humidity (RH) of 70 ± 5%. The colonies of *D. suzukii* were reared on a maize flour-based artificial diet in 50 mL plastic centrifuge tubes (height: 12 cm; diameter: 3 cm) [22]. The artificial diet contained 1000 mL of water, 15 g/L of yeast, and 21 g/L of agar, supplemented with 90 g/L of sucrose, 180 g/L of semolina flour, and 15 g/L of raisins [23]. Twice a week, adult flies were inserted into plastic tubes containing 25 mL of the diet, closed at the top by a fine mesh, and maintained for 2–3 days to encourage oviposition. *T. drosophilae* colonies were reared on *D. suzukii*. The population was maintained on a diet infested by *D. suzukii* larvae and pupae. Adult parasitoids were removed after two weeks just before the new generation emerged. Adults were fed a 10% honey solution in a soaked cotton ball [24].

Temperature and relative humidity inside all climate cabinets were logged at 30 min intervals by a COS-03 Temp/RH system (Shandong Renke Control Technology Co., Ltd., Jinan, China).

### 2.2. Functional Response at Constant Parasitoid Density and Varying Host Densities

To assess the functional response of *T. drosophilae* host density, we used a round transparent plastic food box (10 cm in height and diameter), created a square hole (4 cm × 4 cm) above the box, and used a 200-mesh screen as a vent. Second instar *D. suzukii* pupae were inserted into the boxes at densities of 5, 10, 15, 20, and 25 host pupae/box. Then, we took a pair of male and female *T. drosophilae*, placed them into an artificial climate room, and added 10% honey water. The temperatures were set to 23 ± 1 °C, 25 ± 1 °C, 27 ± 1 °C, 29 ± 1 °C, 31 ± 1 °C, 33 ± 1 °C, 35 ± 1 °C; the RH was 70 ± 5%; and the photoperiod (L:D) was set to 12:12. After 12 h of stimulation, we placed the *T. drosophilae* into plastic boxes with different densities of *D. suzukii* pupae at 23 °C. Twenty-four hours later, we removed the parasitoids and hosts after parasitism and raised them until the parasitoids emerged. Then, we recorded the number of eclosed pupae and dissected the hosts without parasitoids to observe whether they had been parasitized [25].

### 2.3. Mutual Interference Response of Parasitic Wasps

In order to understand the effect of intraspecific interference between parasitoids on the production of progeny, a certain number of host pupae were exposed to different parasitoid densities. We used a round transparent plastic food box (10 cm high and 10 cm in diameter). Then, we opened a 4 cm × 4 cm square hole above the box and created a 200-mesh screen as a ventilation hole. Two-day-old pupae of D. suzukii were then inserted at a density of 50 host pupae/box. After 24 h of eclosion, the T. drosophilae was taken and stimulated for 12 h in artificial climate boxes at different temperatures (23 ± 1 °C, 27 ± 1 °C, 29 ± 1 °C, 31 ± 1 °C, 33 ± 1 °C, 35 ± 1 °C). T. drosophilae wasps (1, 2, 3, 4, and 5 pairs) at different densities were respectively placed into the plastic boxes containing D. suzukii pupae at 23 °C. Twenty-four hours later, the parasitoids were removed and the pupae of D. suzukii were raised until parasitoids emerged [26]. The dead hosts were dissected to observe whether they had been parasitized [27]. The emergence of parasitoids and D. suzukii was observed and recorded. Five replicates were set for each density treatment. For each experiment, the RH was 70 ± 5%, and the photoperiod (L:D) was 12:12.

### 2.4. Data Analysis

The type of predation functional response was determined according to logistic regression analysis between prey density and captured food [28]. The shape (type) of functional response was determined by the maximum likelihood estimation of the parameters of a cubic logistic regression model between the proportion of *D. suzukii* pupae attacked and host pupae density. The response type was determined by the sign of the linear term coefficient of the model. A negative or positive coefficient was considered to be indicative of type II or type III responses, respectively. IBM SPSS Statistics 20.0 and Origin 2017 software were used for statistical calculation and mapping; after the normal distribution test, ANOVA and the LSD method were used for multiple comparative analysis.

The following formula was used [29]:

$$\frac{N_{e}}{N_{0}}=\frac{\exp(P_{0}+P_{1}N_{0}+P_{2}{N_{0}}^{2}+P_{3}{N_{0}}^{3})}{1+\exp(P_{0}+P_{1}N_{0}+P_{2}{N_{0}}^{2}+P_{3}{N_{0}}^{3})}$$

where *N*_0_ represents the initial number of pupae and *N_e_* represents the number of pupae that were parasitized. *P*_0_, *P*_1_, *P*_2_, and *P*_3_ represent constant, first, second, and third power coefficients. If *P*_1_ = 0, then the number of parasitic events rose linearly with increased pupae density; this was considered a Holling I type response. If *P*_1_ < 0 and the number of parasitic events increased at a decreasing rate with increasing pupae density, then this represented a stationary state and was considered a Holling II type response. If *P*_1_ > 0, then the number of parasitic events changed in a sigmoidal way as a function of the density of pupae; this was considered to belong to a Holling III type response [30,31].

The model for the Holling Ⅱ disc equation adjusted to the parasitization data was as follows [32]:

$$N_{a}=aNT/(1+aThN)$$

where *a* represents the instantaneous attack rate, *Th* represents the handling time, *N_a_* represents the daily parasitic amount, *N* represents the prey density and *T* represents the test time (1 d).

The search effect (*S*) proposed by Holling (1959) [17] was fitted with the host density model, as follows:

S=a/(1+aThN)

*a*, *Th*, and *N* were the same as for the Holling Ⅱ equation.

In order to estimate the parameters of the parasitic functional response of *T. drosophilae* in a population, the following modification was made to the random parasitoid equation to include the potential interference among parasitic wasps [33]:

$$A={QP}^{-m}$$

where *A* represents the parasitized number, *P* represents the parasitoid density, *Q* represents the quest constant, and *m* represents the mutual interference constant. The nonlinear least squares regression of the number of parasitized host pupae and parasitoid density was used to estimate the parameters of this functional response modified type II model.

## 3. Results

### 3.1. Functional Response at Constant Parasitoid Density and Varying Host Density after Stimulation at Different Temperatures

**Figure 1:**
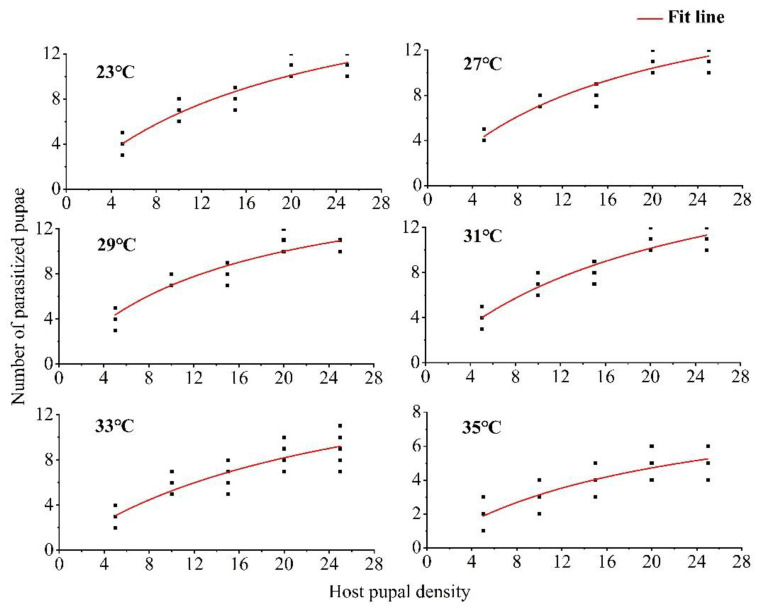
Functional responses of *Trichopria drosophilae* to *Drosophila suzukii* pupae at different temperatures.

According to the logistic regression analysis of the proportion of *Drosophila suzukii* pupae parasitized in relation to the density of pupae, the estimated coefficient of the linear term was always negative. The *P*_1_ values of the control temperature and five treatments at different temperatures were all less than 0, thus indicating that the parasitic functional response type of *T. drosophilae* under different high-temperature stimuli was Holling type II (Table 1). Under different temperature conditions, the Pseudo-R^2^ of each index was very close to 1. This indicated that parasitism increased with host density to an upper asymptote representing the maximum daily per capita reproductive capacity of 1-day-old *T. drosophilae* females (Figure 1).

The instantaneous search rate first increased and then decreased with increasing temperature stimuli. After treatment at 29 °C, the instantaneous search rate reached a maximum of 1.1611, and then decreased with increasing temperature; the lowest instantaneous search rate of 0.4652 was recorded at 35 °C. Within the ranges of 23–29 °C and 31–35 °C, the handling time increased with increasing temperature. The theoretical maximum parasitic value was 20.88 at 31 °C and 9.59 at 35 °C. The parasitic efficiency decreased with increasing temperature, reaching a maximum of 21.73 at 27 °C and a minimum of 4.46 at 35 °C (Table 2).

**Figure 2:**
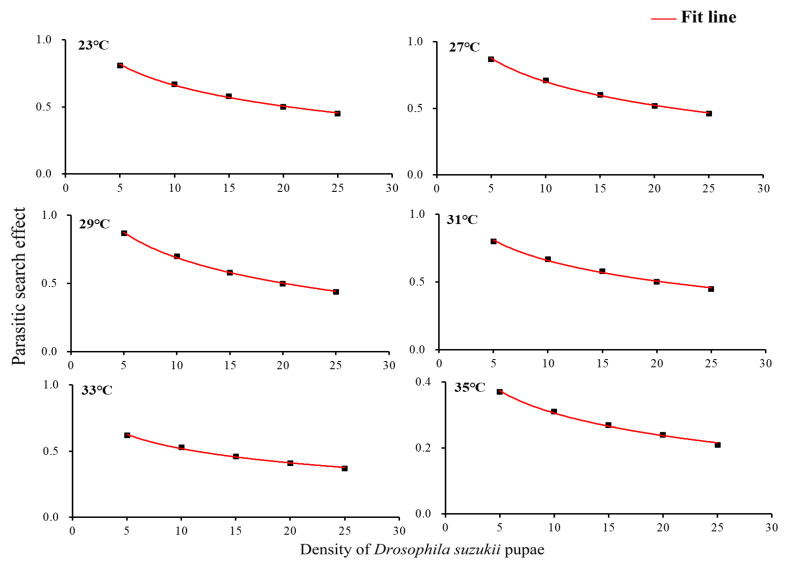
Searching effect of *Trichopria drosophilae* for different densities of *Drosophila suzukii* pupae.

The searching effect of *T. drosophilae* at each temperature gradually fell with increasing prey density from 5 to 25 (Figure 2).

Furthermore, with the same density of *D. suzukii* pupae, the search effect of *T. drosophilae* increased as the temperature decreased. At a pupal density of 5, the search effect of *T. drosophila* stimulated at 27 °C, 29 °C, and 31 °C was significantly higher than at 33 °C and 35 °C (*F*_5,24_ = 5.11, *p* = 0.0001) (Figure 2).

### 3.2. Mutual Interference at Constant Host Density and Varying Parasitoid Densities

In addition, we investigated whether parasitic wasps exhibited different parasitism levels at the same host density, and there were significant differences between treatment groups under different stimulation temperatures (Table 3). When the density of *T. drosophilae* was one pair, the parasitism of *D. suzukii* pupae after stimulation at 35 °C was 9.0; this was significantly lower than other treatments and controls (*F*_5,24_ = 62.21, *p* = 0.0001). When the density of *T. drosophila* was two pairs, the parasitism of *D. suzukii* pupae when stimulated at 31 °C was 18.6; this was significantly higher than other treatment groups (*F*_5,24_ = 80.72, *p* = 0.0001). When the density of *T. drosophila* was three pairs and four pairs, the parasitism of *D. suzukii* pupae when stimulated at 29 °C and 31 °C was significantly higher than that of other treatment groups (three pairs: *F*_5,24_ = 39.64, *p* = 0.0001, four pairs: *F*_5,24_ = 7.68, *p* = 0.0001). When the density of *T. drosophilae* was five pairs, the parasitism of *D. suzukii* pupae when stimulated at 33 °C and 35 °C was significantly lower than that of other treatment groups (*F*_5,24_ = 8.33, *p* = 0.0001).

The average number of *D. suzukii* pupa parasitized (i.e., the number of parasitoid progeny produced) per single *T. drosophilae* female decreased as the stimulation temperature increased; this reduction in the number of pupa parasitized indicated mutual interference among parasitoids (Table 3). Within each temperature treatment group, the parasitism of one pair of parasitic wasps was significantly higher than that of the other treatments (23 °C: *F*_4,20_ = 97.05, *p* = 0.0001, 27 °C: *F*_4,20_ = 97.05, *p* = 0.0001, 29 °C: *F*_4,20_ = 91.45, *p* = 0.0001, 31 °C: *F*_4,20_ = 88.87, *p* = 0.0001, 33 °C: *F*_4,20_ = 94.12, *p* = 0.0001, 35 °C: *F*_4,20_ = 72.68, *p* = 0.0001).

The Hassell–Varley interference model was used to fit the parasitic effects of *T. drosophilae* under different temperature stimuli on its own density interference.

This model can reflect the self-interference of *T. drosophilae* during parasitism. The search coefficient *Q* showed a trend to first increase but then decrease with increasing temperature, reaching a maximum of 19.88 at 31 °C, and the mutual interference coefficient *m* reached a maximum of 0.8923 at 35 °C (Table 3). This indicated that within this temperature range, as the temperature increased, the search ability and mutual interference of *T. drosophilae* also increased. However, when the temperature exceeded 31 °C, the interference exceeded its increased search ability.

## 4. Discussion

### 4.1. Functional Response after Stimulation at Different Temperatures

Functional response is an important indicator for evaluating the parasitism or predation efficiency of the natural enemies of pests [34]. Since Solomon [35] first proposed the functional response method, parasitic functional response has become an important standard for measuring the effect of parasitic wasps in pest control [36]. The functional response type is a basic element that is used to understand the relationship between host density and the number of hosts parasitized [37]. Although laboratory studies on functional responses have little similarity to field contact hosts, they provide useful information relating to the potential efficacy of biological control agents. The rational use of functional response models for parasitic natural enemy insects can significantly improve the efficiency of using parasitic natural enemy insects to control pests in the field [38]. Our present results indicate that within the temperature range of the experiment, the parasitic functional response type of *Trichopria drosophilae* to *Drosophila suzukii* pupae conforms to the Holling II functional model. The parasitic effect increased with increasing host density, although the increase in parasitic effect slowed down when the host density increased to 20. This is consistent with the Holling II results of the study conducted by Fang et al. (2019) [31] on the functional response of *T. drosophilae* at 23 °C and also shares the same type of parasitic wasp functional response with *Leptopolina japonica* [39]. This may be related to the limited number of mature eggs per unit time of the parasitoid [40]. However, this is inconsistent with the results of Kacar et al., who found that the type of parasitic functional response of *T. drosophilae* to *D. suzukii* and *D. melanogaster* pupae is a linear function [16]. Their *T. drosophilae* was collected in the wild in California, USA. Our *T. drosophilae* collection is similar to that of Yunnan Province in China, which may be due to the inconsistency of parasitic functional response types caused by different geographical populations.

We found that the instantaneous attack rate of *T. drosophilae* was the highest at 29 °C and the lowest at 35 °C. Within the ranges of 23–29 °C and 31–35 °C, the handling time increased with increasing temperature. This is consistent with the pattern discovered by Li et al. [41] in their study of the effect of temperature on the functional response of *Spalangia endius* to the pupae of *Bactrocera curubitae*. These authors found that adverse environments promoted the reproductive potential of parasitic wasps to produce offspring. The reduction in handling time within the range of 29–31 °C is inconsistent with the findings of Chen et al. [42] relating to the functional response of *Encarsia guadeloupae* Viggiani on *Aleurodicus dispersus* Russell; this may be related to the physiological indicators of energy metabolism substances and physiological enzyme activity in the body at different temperatures. Several studies have evaluated the effects of temperature on the energetic efficiency of parasitic wasps: some studies found that the energetic efficiency of parasitic wasps decreased with increasing temperature [43], although a reverse relationship was reported by Vasseur and McCann [44]. At the same host density, the parasitism efficiency of *T. drosophilae* was the highest at 27 °C and the lowest at 35 °C; this was consistent with the findings reported by Wang et al. [45] after investigating the parasitic functional response of *Parasitalis tenuis* to *Spodoptera frugiperda*. In the present study, the parasitic efficiency of the parasitoids first increased and then decreased with increasing temperature, thus indicating that temperature was an important factor affecting the parasitic efficiency of *T. drosophilae* against *D. suzukii* pupae. When breeding *T. drosophilae* indoors, it is necessary to control the environmental temperature efficiently. At a temperature of 31 °C and a pupal density of 50, more pupae could be parasitized, and when releasing parasitic wasps into the field, it is necessary to choose a suitable environmental temperature and measure the density of *D. suzukii* in the field to maximize their parasitic potential.

### 4.2. The Search Effect and Interference Effect of Trichopria drosophilae

Our analysis showed that the search effect decreased as host density increased but increased with increasing temperature within the test temperature range; the highest search effect was recorded at 29 °C. Simulation results relating to the *T. drosophila* self-interference effect model showed that when the parasitic space and host density were fixed, the number of hosts that could be parasitized by female wasps did not increase proportionally with the increase in the parasitic wasp’s own density, thus indicating that interference effects exist among *T. drosophilae* individuals. Furthermore, within a certain spatial range, the interference effect increased with increasing parasitic wasp density. Therefore, in the breeding and field application of parasitic wasps, the number of parasitic wasps should be reasonably determined according to the size of the space and host density, so as to reduce the interference between parasitic wasps and reduce the cost while ensuring a high parasitic rate [46]. The density of *T. drosophilae* itself will also have an obvious impact on its parasitic effect. The results showed that *T. drosophilae* had an interference effect on *D. suzukii* pupae. The average parasitic effect of *T. drosophilae* decreased as its own density increased. The cause of intraspecific interference by *T. drosophilae* may be related to competition among individuals due to high density [47]. The intraspecific competition of species can be divided into two forms [48]: competition and sharing competition. When *T. drosophilae* parasitized *D. suzukii* pupae, this involved sharing competition and all parasitized wasps had equal opportunities to approach their prey. When the number of *D. suzukii* pupae was fixed, the competition intensity of *T. drosophilae* increased with increasing density; this caused an increase in intraspecific interference.

In conclusion, under laboratory conditions, in the temperature range of 23 °C to 35 °C, *T. drosophilae* showed a type II functional response. At 31 °C, the theoretical parasitic maximum of *T. drosophilae* reached a maximum, and expanding the reproductive effect is the best in this environment. When the temperature is 31 °C, the parasitoid:host pupa = 1:50, and the theoretical release effect is the best.

## 5. Conclusions

Under laboratory conditions, *T. drosophilae* showed good ability to parasitize *D. suzukii* pupae. In fact, in the temperature range of 23 °C to 35 °C, *T. drosophilae* showed a type II functional response. At 31 °C, the theoretical parasitic maximum of *T. drosophilae* reached a maximum, and expanding the reproductive effect is the best in this environment. When the temperature is 31 °C, the parasitoid:host pupa = 1:50, and the theoretical release effect is the best.

## Figures and Tables

**Table 1:** Verification of the parasitic model for *Trichopria drosophilae* at different temperatures (23–35 °C).

Temperature (°C)	Coefficient	Estimate	SE	Pseudo-R^2^
23	*P*_1_	−0.89	0.40	0.90
27	*P*_1_	−1.39	0.48	0.93
29	*P*_1_	−1.57	0.60	0.91
31	*P*_1_	−0.48	0.29	0.91
33	*P*_1_	−0.13	0.29	0.79
35	*P*_1_	−0.06	0.22	0.70

**Table 2:** Parasitic functional response formulae of *Trichopria drosophilae* to *Drosophila suzukii* pupa at different temperatures.

Temperature (°C)	Holling II Disc Equation	Instantaneous Search Rate (*a*)	Handling Time (*Th*)	Theoretical Parasitic Maximum (*N_amax_*)	Parasitic Efficiency (*a*/*Th*)	Pseudo-R^2^
23	*Na* = 1.0102*Nt*/(1 + 0.0500*Nt*)	1.0102 ± 0.1118	0.0495 ± 0.0062	20.20	20.41	0.89
27	*Na* = 1.1169*Nt*/(1 + 0.0573*Nt*)	1.1169 ± 0.1094	0.0514 ± 0.0050	19.46	21.73	0.90
29	*Na* = 1.1611*Nt*/(1 + 0.0662*Nt*)	1.1611 ± 0.1292	0.0570 ± 0.0056	17.54	20.37	0.88
31	*Na* = 0.9886*Nt*/(1 + 0.0474*Nt*)	0.9886 ± 0.1001	0.0479 ± 0.0057	20.88	20.64	0.91
33	*Na* = 0.7384*Nt*/(1 + 0.0401*Nt*)	0.7384 ± 0.1191	0.0543 ± 0.0119	18.42	13.59	0.80
35	*Na* = 0.4652*Nt*/(1 + 0.0485*Nt*)	0.4652 ± 0.0907	0.1043 ± 0.0234	9.59	4.46	0.72

**Table 3:** Parasitism rate of *Drosophila suzukii* pupa and self-interference response.

Temperature	Number of *Trichopria drosophilae* (pair)	Total Number of Parasitic	Parasitism Rate	Interference Equation	Pseudo-R^2^
23 °C	1	18.6 ± 0.51 Aa	37.2 ± 1.02%	*A* = 19.78P^−0.6921^	0.76
	2	15.4 ± 0.51 ABb	30.8 ± 1.02%		
	3	13.4 ± 0.68 Ab	26.8 ± 1.36%		
	4	7.5 ± 0.68 Ac	15.0 ± 1.36%		
	5	5.2 ± 0.37 Ad	10.4 ± 0.75%		
27 °C	1	17.6 ± 0.51 Aa	35.2 ± 1.02%	*A* = 18.76P^−0.6115^	0.76
	2	14.4 ± 0.51 ABb	28.8 ± 1.02%		
	3	12.4 ± 0.68 ABb	24.8 ± 1.36%		
	4	6.6 ± 0.68 Ac	13.2 ± 1.36%		
	5	4.2 ± 0.37 Ac	8.4 ± 0.75%		
29 °C	1	16.4 ± 0.51 Aa	32.8 ± 1.02%	*A* = 17.28P^−0.6406^	0.83
	2	13.0 ± 0.71 Bb	26.0 ± 1.41%		
	3	10.0 ± 0.32 Bb	20.0 ± 0.65%		
	4	6.2 ± 0.58 ABc	12.4 ± 1.17%		
	5	4.2 ± 0.37 Ad	8.4 ± 0.75%		
31 °C	1	18.6 ± 0.51 Aa	37.2 ± 1.02%	*A* = 19.88P^−0.5622^	0.75
	2	15.8 ± 0.37 Ab	31.6 ± 0.75%		
	3	13.4 ± 0.68 Ab	26.8 ± 1.36%		
	4	7.8 ± 0.86 Ac	15.64 ± 1.72%		
	5	5.2 ± 0.37 Ad	10.4 ± 0.75%		
33 °C	1	17.2 ± 0.37 Aa	34.4 ± 0.75%	*A* = 18.37P^−0.6878^	0.80
	2	14.4 ± 0.25 ABb	28.8 ± 0.51%		
	3	9.8 ± 0.37 Bc	19.6 ± 0.75%		
	4	6.0 ± 0.84 ABd	12.0 ± 1.68%		
	5	3.6 ± 0.81 ABd	7.2 ± 1.62%		
35 °C	1	9.0 ± 0.31 Ba	18.0 ± 0.62%	*A* = 9.048P^−0.8923^	0.91
	2	4.8 ± 0.20 Cb	9.6 ± 0.40%		
	3	4.0 ± 0.55 Cbc	8.0 ± 1.10%		
	4	2.6 ± 0.25 Bcd	5.2 ± 0.51%		
	5	1.6 ± 0.25 Bd	3.2 ± 0.51%		

Different uppercase letters in the table indicate significant differences in the parasitic effect of *Trichopria drosophilae* under the same host density after stimulation at different temperatures (LSD, *p* < 0.05). Different lowercase letters indicate significant differences in the parasitic effect of *Trichopria drosophilae* under different host densities at the same temperature (LSD, *p* < 0.05).

## Data Availability

The datasets analyzed in the present study are available from the corresponding authors on reasonable request.
